# 5Gs for crop genetic improvement

**DOI:** 10.1016/j.pbi.2019.12.004

**Published:** 2020-08

**Authors:** Rajeev K Varshney, Pallavi Sinha, Vikas K Singh, Arvind Kumar, Qifa Zhang, Jeffrey L Bennetzen

**Affiliations:** 1 Center of Excellence in Genomics & Systems Biology, International Crops Research Institute for the Semi-Arid Tropics (ICRISAT), Hyderabad, 502324, India; 2 International Rice Research Institute, South Asia Hub, ICRISAT, Hyderabad, 502324, India; 3 IRRI South Asia Regional Center, NSRTC Campus, G.T. Road, Collectry Farm, P.O. Industrial Estate, Varanasi, 221006, India; 4 National Key Laboratory of Crop Genetic Improvement, Huazhong Agricultural University, Wuhan, 430070, China; 5 Department of Genetics, University of Georgia, Athens, GA 30602, USA

## Abstract

Here we propose a 5G breeding approach for bringing much-needed disruptive changes to crop improvement. These 5Gs are Genome assembly, Germplasm characterization, Gene function identification, Genomic breeding (GB), and Gene editing (GE). In our view, it is important to have genome assemblies available for each crop and a deep collection of germplasm characterized at sequencing and agronomic levels for identification of marker-trait associations and superior haplotypes. Systems biology and sequencing-based mapping approaches can be used to identify genes involved in pathways leading to the expression of a trait, thereby providing diagnostic markers for target traits. These genes, markers, haplotypes, and genome-wide sequencing data may be utilized in GB and GE methodologies in combination with a rapid cycle breeding strategy.

## Highlights

• 5G breeding approach brings precision and enhances efficiency in breeding programs.
• Genome, germplasm, gene function, genomic breeding and genome editing are the 5Gs.
• NGS platforms, speed breeding and express edit facility are the key drivers for 5G breeding.
• Haplotype-based breeding, genomic selection and gene editing are the key genomic breeding approaches of the future.
• Multi-disciplinary team of scientists need to be trained to deploy 5G breeding in developing countries.

**Current Opinion in Plant Biology** 2020, **56**:190–196

This review comes from a themed issue on **AGRI**

Edited by **David Edwards**

For a complete overview see the Issue and the Editorial

Available online 28th January 2020

**https://doi.org/10.1016/j.pbi.2019.12.004**

1369-5266/© 2019 The Author(s). Published by Elsevier Ltd. This is an open access article under the CC BY license (http://creativecommons.org/licenses/by/4.0/).

## Introduction

Dramatic and rapid climate change will cause extreme weather, including droughts, floods and other disasters. Food production will suffer greatly from these changes. The nearly 80 percent of the world’s population that are poor and live in rural areas typically rely on local agriculture for their survival [1]. It has been predicted that, on average, global yields of major crops will be reduced 6.0% in wheat, 3.2% in rice, 7.4% in maize, and 3.1% in soybean for every degree Celsius increase in global mean temperature [2]. In this regard, the CGIAR system (https://www.cgiar.org/) initiated a ‘Two Degree Initiative for Food and Agriculture’. This initiative is targeted on assisting ∼200 million small scale food producers across the globe to adapt at the speed and scale needed for the current pace of climate change. Improving access to climate-smart technologies and practices, including this development of climate-resilient high yielding varieties and their rapid availability to farmers’ fields, will provide an opportunity to achieve climate smart solutions [3].

**Figure 1:**
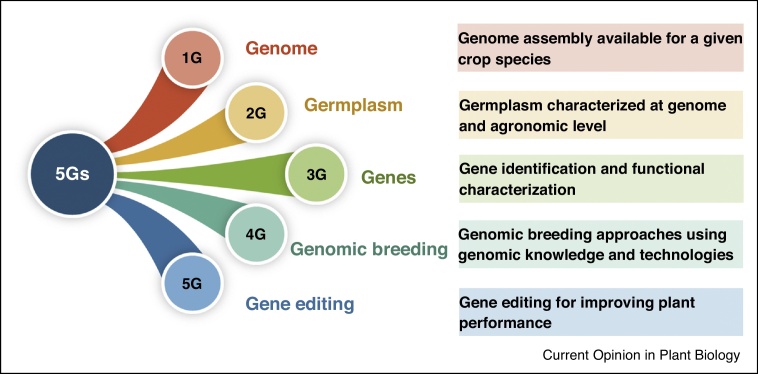
5Gs for crop genetic improvement.

Crop improvement for food and nutritional security, especially in the context of continuous population growth and such challenges as climate change and water scarcity, have become important global concerns [4^••^]. Facing these threats, current crop breeding strategies will not yield a sufficient rate of crop improvement to meet demands in the short-term or long-term future. Hence, we propose a 5G breeding strategy to dramatically accelerate crop genetic improvement. The 1st G is *G*enome assembly for each crop species, the 2nd G is *G*ermplasm characterized at genomic and agronomic levels, the 3rd G is *G*ene function identification, the 4th G is *G*enomic breeding methodologies, and the 5th G is *G*ene editing technologies (Figure 1).

In the following sections, we describe the 5Gs for enhancing crop improvement. We conclude with a discussion of the current challenges and opportunities for integrating the 5Gs into crop improvement.

## 1st G: Genome assembly

Advances in next-generation sequencing (NGS) technologies coupled with improved genome assembly algorithms have facilitated the *de novo* assemblies of >264 plant genomes, including such crops as rice, maize, wheat, barley, soybean, cotton, sorghum, tomato, pigeonpea, chickpea, and groundnut. The quality of these genome assemblies varies tremendously, from nearly finished genomes to draft genomes with hundreds of unoriented sequence scaffolds. A few meet the platinum genome standard, including assemblies with full-chromosome scaffolds and haplotypes resolved across the entire genome, preferably including strong links to the genetic map. However, most plant genome assemblies are draft genomes. Recent advancements in sequencing technologies, particularly long read generation and physical map linkages, can now often generate chromosome-scale, fully phased diploid genome assemblies for any species at the platinum genome level [5].

The availability of a genome assembly provides an opportunity to develop genomics tools and technologies for such applications as trait discovery and molecular breeding. All genetic variation can be described, including SNPs, insertions, deletions, transversions, copy number variations and epigenetic changes [6]. These variants are useful in the development of customized SNP arrays [7], that can be utilized for development of saturated genetic maps and QTL identification. Sequence variant information defines haplotypes [8], which can then be employed for overcoming or taking advantage of linkage disequilibrium in a breeding program [9]. Genome assembly information is also vital for developing a gene expression atlas, proteome maps, metabolome maps, and epigenome maps.

With the ongoing and deep reductions in sequencing costs, large-scale re-sequencing projects have been initiated in several crops. For instance, 3010, 994 and 429 germplasm accessions have been re-sequenced in rice [10^••^], pearl millet [11^••^], and chickpea [12], respectively. Such projects generate ‘big data’ that pose storage and computational challenges. These challenges include compilation, curation, complex data analyses, visualization, retrieval and sharing [13]. To accelerate use of genome sequence information in next-generation breeding, customized informatics platforms are needed. In this context, some initiatives/platforms such as SNPSeek (for rice) (https://snp-seek.irri.org/_snp.zul), Genomic Open-source Breeding Informatics Initiative (GOBII) (http://cbsugobii05.tc.cornell.edu/wordpress/) and Excellence in Breeding Platform (EiB) (https://excellenceinbreeding.org/) have become available. These platforms will be vital to breeders for mining superior alleles/haplotypes, thus identifying the most-suitable parental lines for breeding populations.

## 2nd G: Germplasm characterization

During the course of crop domestication and breeding, cultivar genetic diversity is narrowed for all traits [14], but national and international ‘genebanks’ (germplasm repositories/germplasm banks) provide a rich source of diverse alleles that may be vital for future crop improvement. The ∼1750 plant germplasm banks worldwide hold ∼7.4 million accessions (www.fao.org), but <2% of these materials have been used as plant genetic resources (PGRs), although these few uses have led to major crop improvements [15^••^]. One of the reasons behind this limited use of PGRs is the overwhelming number of accessions that have no trait or other genetic information. Therefore, we propose characterization of as many accessions as possible at both genomic and agronomic levels. If the phenotyping is performed at specific nursery locations, and with community-established criteria, the provided information will allow deep genome wide association studies (GWAS) and identification of GXE effects. This provides the information to determine the potential agronomic value of particular alleles and accessions that will allow informed decision-making in breeding programs.

While NGS-based approaches have allowed comprehensive sequencing of large germplasm collections in several crops, field phenotyping lags dramatically. For instance, whole-genome re-sequencing (WGRS) has investigated 3010 rice accessions [10^••^], while genotyping-by-sequencing (GBS) has been utilized to characterize 44 624 wheat breeding lines [16^•^] and 20 000 wild and domesticated barley accessions [17]. These studies are initial examples of how genomics and informatics technologies can characterize large crop germplasm collections [18]. These studies are providing genome-wide variant information and insights on population structure, crop domestication, and so on. However, for mining useful genetic information, it is imperative to phenotype the collections. NGS technologies together with some phenotyping have been utilized in a few crops for identification of marker-trait associations, including rice [19^•^], foxtail millet [20], pigeonpea [21^•^], pearl millet [11^••^], cotton [22], rapeseed [23], chickpea [12] and grape [24]. These studies have provided information on the genetic architecture of agriculturally important traits and the identification of valuable alleles for morphological, agronomic, developmental and quality-related traits. In the future, sequencing of entire germplasm collections present in genebanks and association with phenotypes should be a primary component for all crop-breeding programs.

Large-scale germplasm characterization also provides information on the presence of haplotypes at a particular locus for a given trait that can be used in haplotype-based breeding strategies ([25^••^], see later) or the genomic selection approach. Similarly, deleterious effect mutations (genetic load) can also be identified [26], and then can be purged by marker-assisted selection or gene editing, as suggested by Johnsson *et al.* [27^•^]. Eventually, superior parental lines will be identified with the best alleles at each locus, including minimum genetic load, and introduced into breeding programs with a plan to optimize the best allelic combinations. As an early step towards this optimal goal, current haplotype information can be used to select parents for nested association mapping (NAM) and multi-parent advanced generation inter-cross (MAGIC) populations for high-resolution gene:trait discovery.

Recent advances in genomics have led to the development of various sequencing-based rapid trait mapping approaches such as BSR-Seq [28], MutMap [29], QTLseq [30] and Indel-seq [31]. NGS technologies have enabled modification and improvement of traditionally tricky, time-consuming bulked segregant analysis (BSA, [32]) into rapid and whole-genome sequence-based high-resolution trait mapping [33^•^]. Due to the availability of genome assemblies, inexpensive high-throughput WGRS pipelines have become available, so that the use of sequence-based trait mapping approaches has become possible in several crop species. Following this approach, sequencing-based trait mapping can be broadly grouped into two classes: i) trait mapping through pooled sequencing of populations, and ii) trait mapping through complete sequencing of populations. Several examples of NGS-based trait mapping have been reported in crops [34]. This kind of trait mapping has several advantages over traditional marker-based mapping. For instance, in addition to taking much less time, these approaches identify genes or even quantitative trait nucleotides (QTN) for a given trait. In several cases, such QTNs have been converted into diagnostic markers. We believe that genes and markers identified by using these approaches will have a uniquely high prediction/diagnostic power for breeding applications.

## 3rd G: Gene function identification

Using a range of functional genomics and trait mapping approaches, a large number of candidate genes with associated molecular markers for traits of interest have been identified in many crops. For instance, various -omics platforms were established in the past that have allowed the functional characterization of about 2296 genes controlling major traits in rice [35,36]. However, in most crops, the great majority of candidate genes, identified through transcriptomic approaches and/or mapping, are far from confirmation. Moreover, the molecular mechanisms of their potential agronomic values need to be understood in detail.

Systems Biology is an emerging holistic approach that proposes full understanding of biological systems by combining -omics approaches such as genomics, transcriptomics, epigenomics, proteomics, and metabolomics, together with modeling and high-performance computational analysis [37^•^]. In brief, systems biology is the study of an organism and/or trait, viewed as an integrated and interacting network of genes, proteins, and biochemical reactions, including the inputs from various internal and external environments. One goal of systems biology is to discover emergent properties derived from molecular interactions that will further our understanding of the entirety of processes that occur in a biological system. In furtherance of this goal, gene expression atlases [38, 39, 40, 41, 42], epigenome maps [43, 44, 45], proteome maps [46, 47, 48] and metabolome maps [49, 50, 51] have been developed in some crop species. Availability of these resources will accelerate the use of systems biology approaches to understand the molecular mechanism of complex traits such as drought tolerance [52] or heterosis [53]. Once traits are associated with particular pathways, and superior alleles identified, then breeders can employ a deeper understanding of plant biology to predict parental and allelic combinations that will uncover improved agronomic traits.

## 4th G: Genomic breeding (GB)

Genomic breeding involves approaches that use multi-omics data, knowledge resources, genes and technologies generated by genomics research for breeding the genomes to enhance crop breeding programmes. [35]. Although some methods of GB such as marker-assisted selection (MAS), marker-assisted backcrossing (MABC) and marker-assisted recurrent selection (MARS) have been used for breeding in several crops, it is important to have GB methodologies well-integrated into most or all crop breeding programs. In addition to above-mentioned GB methodologies, some new approaches such as forward breeding (FB), haplotype-based breeding (HBB) and genomic selection (GS), coupled with speed breeding (SB), have also been suggested for enhancing the precision, efficiency and rate of acquired genetic gain in crop breeding [34]. While diagnostic markers associated with genes and major effect QTL are required for MAS, MABC and FB, superior haplotypes at a given locus for a target trait need to be identified for HBB. The GS approach, in contrast, does not need markers specifically associated with a trait because breeding lines are selected for crossing and advancing generations based on genomic-estimated breeding values calculated from genome-wide marker data.

Considering the breeding objectives, any of above-mentioned GB approaches can be chosen for crop improvement. For example, if breeders need to select parental lines or introgress some major effect QTL for a target trait, MAS and MABC approaches can be used. MABC is useful to introgress a few loci (<10) for improving elite varieties. This approach has been extensively used to develop a large number of breeding lines for commercial release in public and private sectors. The FB approach will be the best option when early generations of segregating populations (e.g. F_2_ generations) are used to advance plants carrying the target QTL/gene. The MARS approach is useful to introgress from 10 to 40 loci through intercrossing elite × elite parents to develop superior lines with an optimum combination of superior alleles [34].

Recent re-sequencing of germplasm collections in a few crops has facilitated identification of a small number of strong marker-trait associations and haplotypes for target traits [54,55]. ‘Haplotype assembly’ was proposed as one new approach for developing improved crops through assembling superior haplotypes of the targeted traits [25^••^]. ‘Superior haplotypes’, in which the phenotypic performance of the group of individuals sharing a haplotype (‘specific haplotype group’), can be identified. The identified superior haplotypes then can be utilized in the breeding program through haplotype-assisted breeding.

GS is an approach using genome-wide selection with a large number of markers [56]. GS works upon defined ‘genomic estimated breeding values’ (GEBVs) that are calculated from the genotypic and phenotypic dataset of a ‘training population.’ This approach has a higher accuracy of prediction of elite genetic materials in the initial generations and permits shorter breeding cycles. GS, reviewed by Crossa *et al.* [57^•^], has been extensively used in several crops. Very recently, Watson *et al.* [58^••^] introduced the concept of ‘speed breeding’ by giving plants light for 22 hours and dark for only 2 hours. Speed breeding shortens generation times, and thus has been proposed or is now being used for many crops [59]. In fact, speed breeding has also been suggested to be coupled with GS in a process called SpeedGS, for rapid development of new breeding lines [1]. GS combined with superior haplotypes (Haplo-GS) is another new and promising approach for the rapid development of new breeding lines.

## 5th G: Gene editing (GE)

GE has emerged as a powerful approach for improving plant performance and the development of various abiotic and biotic stress tolerance lines. With the recent discovery of *Cas9* guide RNA and availability of functional genomics data coupled with advances in bioinformatics pipelines, targets are being identified and subjected to editing. A large number of genes with significant phenotypic effects have been cloned and functionally characterized. As a result, GE has been used to generate useful traits in such crops as rice, maize, wheat, sugarcane, soybean, potato, sorghum, orange, cucumber, tomato, flax, and cassava, for traits like herbicide resistance, drought tolerance, thermo-sensitive genic male sterility, disease resistance and altered product quality, including some in the process of commercial release [60]. For instance, Oliva *et al.* [61] edited promoters of *SWEET11*, *SWEET13* and *SWEET14* at effector-binding elements recognized by the pathogen *Xanthomonas oryza* pv *oryzae*, a causal agent for rice bacterial blight. These experiments generated rice plants that are broadly resistant to the pathogen. To enhance the durability and management of resistance, Eom *et al.* [62] developed a kit to trace the disease, its virulence and resistance alleles. However, the stewardship of gene-edited lines in combination with an appropriate deployment strategy is essential to meet environmental health and safety standards. There remains a lack of clarity as to the GMO or non-GMO status of such germplasm in many countries [63]. It is anticipated that legislation and a better-educated public will soon allow the benefits of this research to reach the farming community [64].

It is also important to mention that the GE approach is not only useful to create novel alleles, it can also be used for the promotion of superior alleles [65] and removal of deleterious effect alleles [27^•^] identified through large-scale sequencing efforts. Furthermore, it has been suggested that a reverse domestication approach could be pursued for new crops or current crops by editing genes related to domestication traits in wild species. This could provide crop diversification and make available superior lines with enhanced stress resistances. As this approach may require several cycles of editing and line fixation, ‘ExpressEdit’ approaches that combine speed breeding with GE have been suggested [1].

## Conclusions and prospects

Although components of the described 5Gs are being used in public and private crop improvement programs in several developed countries, comprehensive 5G integration is lacking, especially in developing countries. However, we are hopeful that recent advances in sequencing, phenotyping and data science will accelerate utilization of the 5G strategy in coordinated crop improvement programs worldwide. In this context, capacity building of young scientists in developing countries is required in 5G breeding to handle, analyze and interpret the enormous data sets from sequencing, genotyping, phenotyping, -omics and systems biology studies pursued across large-scale germplasm collections. In particular, training on breeder-friendly pipelines, analytical and decision support tools and databases related to identification of variants and haplotype, diversity analysis, sequencing-based trait mapping, identification of GE targets and implementation of GB methodologies will be very helpful. In summary, a comprehensively applied 5G breeding can enhance the precision, efficiency and effectiveness of breeding programs to develop climate-resilient, high-yielding and nutritious varieties while delivering a high rate of genetic gain in any breeding program, including in developing countries where these gains are most needed.

## Conflict of interest statement

Nothing declared.

## References and recommended reading

Papers of particular interest, published within the period of review, have been highlighted as:

• of special interest
•• of outstanding interest
